# Random access with a distributed Bitmap Join Index for Star Joins

**DOI:** 10.1016/j.heliyon.2020.e03342

**Published:** 2020-02-17

**Authors:** Jaqueline J. Brito, Thiago Mosqueiro, Ricardo R. Ciferri, Cristina D.A. Ciferri

**Affiliations:** a University of São Paulo, São Carlos, Brazil; b University of California Los Angeles, Los Angeles, USA; c Federal University of São Carlos, São Carlos, Brazil

**Keywords:** Computer science, Random access, Distributed Bitmap Index, Star Join, Low-selectivity queries, Hadoop ecosystem

## Abstract

Indices improve the performance of relational databases, especially on queries that return a small portion of the data (i.e., low-selectivity queries). Star joins are particularly expensive operations that commonly rely on indices for improved performance at scale. The development and support of index-based solutions for Star Joins are still at very early stages. To address this gap, we propose a distributed Bitmap Join Index (dBJI) and a framework-agnostic strategy to solve join predicates in linear time. For empirical analysis, we used common Hadoop technologies (e.g., HBase and Spark) to show that dBJI significantly outperforms full scan approaches by a factor between 59% and 88% in queries with low selectivity from the Star Schema Benchmark (SSB). Thus, distributed indices may significantly enhance low-selectivity query performance even in very large databases.

## 1 Introduction

The volume of data that is now available changed the design and value of decision-making systems on a broad range of fields [1], [2], [3]. To harness the true potential behind this new paradigm, the demand for the appropriate infrastructure rapidly inspired novel and creative solutions, such as distributed file systems, parallel programming models, and NoSQL databases. Due to the high costs of maintaining updated computational resources, cloud computing was proposed based on commodity hardware and non-local infrastructures [4], [5]. For instance, Apache Hadoop [6] was released as an open-source framework used for distributed storage and processing (i.e., Hadoop MapReduce) of big datasets. The next generation of technologies such as HBase [7] and Spark [8] were all designed to interact with Hadoop systems. Among other options [9], [10], the emergence of these powerful open-source solutions paved the way for the development of applications that utilize billions of entries to construct reports and analytics [11], [12]. Consequently, there is an increasing demand for flexible and scalable solutions to store and efficiently query large datasets.

Research demonstrated the importance of indices to improve the performance of relational databases, especially to execute queries that return a small portion of the data, i.e., queries with *low selectivity* [13], [14], [15]. As a rule of thumb, low-selectivity queries benefit the most from the use of indices (i.e., picking entry by entry based on their indexing without the need to analyze the whole table), as opposed to full table scan. This also applies to NoSQL databases and applications to Big Data [16], [17]. For example, in the context of credit card transactions, although historical records are often used to generate statistical polls to aid decision-making processes in banks and hedge funds, a share of the profits in such companies result from the evaluation of records involving only a handful of individuals (e.g. loan applications or personalized pricing algorithms).

**Figure 1:**
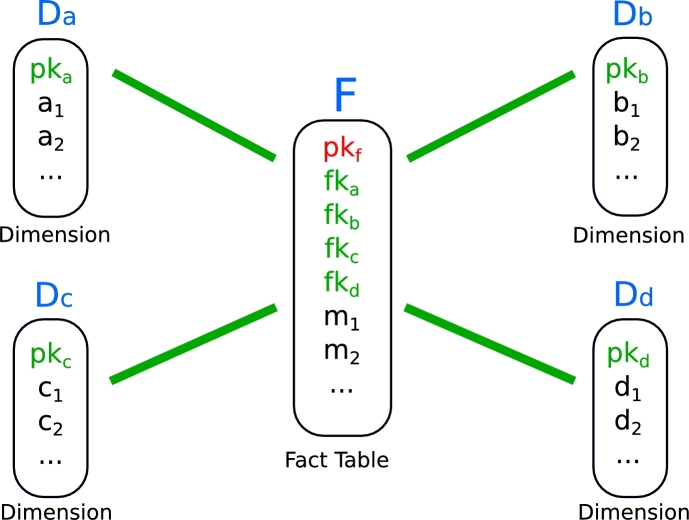
A visualization of the star schema with one central fact table, and four dimension tables.

Star Joins are demanding operations in Online Analytical Processing (OLAP) systems that often present low selectivity even in very large datasets, thus benefiting from indexed solutions [18], [19]. For instance, the Bitmap Join Index distinguishes itself as a largely used solution to improve Star Joins in non-cloud environments [20]. Star Joins are defined on a star schema, where a central fact table is linked to several satellite dimension tables, thus resembling a star (Fig. 1). A large collection of strategies and optimizations have been proposed for Star Joins in cloud environments (see Section 3). For instance, many MapReduce strategies based on full scan were introduced to deal with the rampant growth in data volume [21], [22], [23]. The challenge then became to avoid excessive disk access and cross-communication among parallel jobs [11], [24], [25]. However, as the query selectivity becomes small, a considerable portion of the data retrieved by full scan operations is inevitably discarded, draining read/write resources. Also, shuffling unneeded data clogs the network and blocks further cross-communication.

Still, the development and support of Hadoop-based solutions for random access remain on hold. Particularly, although queries with low selectivity may still require the retrieval of massive amounts of entries, our overarching hypothesis is that the appropriate use of indexing should substantially improve the current methods, depending on the query selectivity. In other words, even in a distributed file system, query selectivity may reach a point where the use of indices to retrieve results entry by entry is faster than scanning and subsequently pruning the fact table. Yet, all of the available solutions to Star Joins in Hadoop use full scan regardless of their selectivity [26], [27]. Moreover, an ideal distributed system should seamlessly switch between full scan and random access according to properties related to the query (e.g., its selectivity).

We propose a strategy that combines distributed indices and a two-layer architecture based on open-source frameworks to accelerate Star Join queries with low selectivity. To propose our strategy, we address the following fundamental *challenges*.

1. *How to provide full scan or random access to speed up Star Joins over fact tables stored in the HDFS according to the query selectivity.* To this end, we base our strategy on a two-layer architecture that delegates massive parallel operations to a Processing Layer, and the access to the distributed file system to an Access Layer (Fig. 2). The use of these two independent layers allows the choice of different processing strategies based on their individual characteristics to solve a broad spectrum of queries, including high and low selectivity.

**Figure 2:**
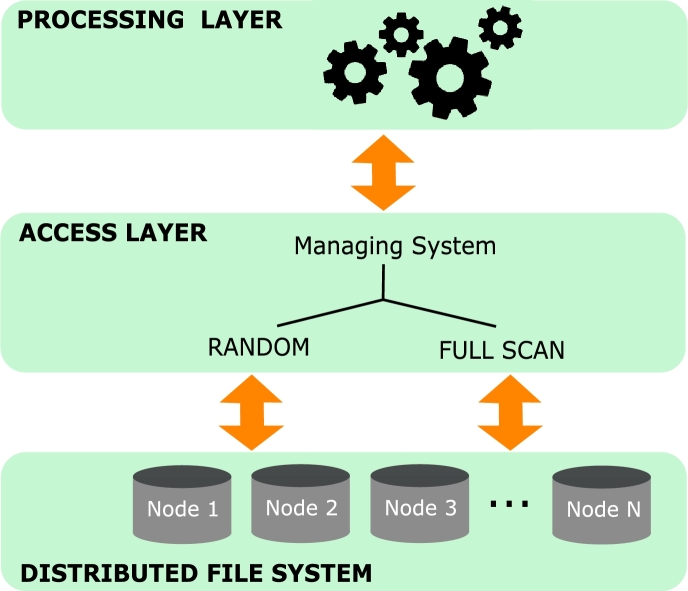
The architecture explored in this paper, with a Processing Layer responsible for massive distributed computation and an Access Layer capable of performing full scan and random access.
2. *What is the suitable distributed data structure to store Bitmap Join indices for large-scale data in the HDFS?* We propose the distributed Bitmap Join Index (dBJI) that is partitioned across a distributed system, and fully exploits the parallel resources available on the cluster. The dBJI can be used for random access in the cloud.
3. *How to design a distributed algorithm that scales well with increasing data volumes and provides a suitable index partitioning.* We propose a distributed algorithm to efficiently construct the dBJI. Our algorithm is characterized by partitioning the index structure across the nodes with a given partition size.
4. *How to design an efficient algorithm for low-selectivity Star Joins using the distributed Bitmap Join index.* We propose an efficient processing algorithm for low-selectivity Star Joins in linear time based on available frameworks designed for cloud systems. The algorithm is divided into two phases: first, the dBJI is used by the Processing Layer to solve the dimension predicates; then the requested primary keys are retrieved by the Access Layer using random access.
5. *How to implement the solutions to the aforementioned challenges using Hadoop-related software.* We instantiated the Access Layer with HBase, and the Processing Layer with either Spark or MapReduce. All implementations are provided on GitHub [28].

The advantages of our index-based solution were investigated through an in-depth performance analysis in low-selectivity Star Joins considering a wide range of related work available in the literature. The performance results showed that our solution outperformed by a factor between 59% and 88% other 11 strategies based on full scan.

## 2 Background

### 2.1 The Bitmap Join Index

**Figure 3:**
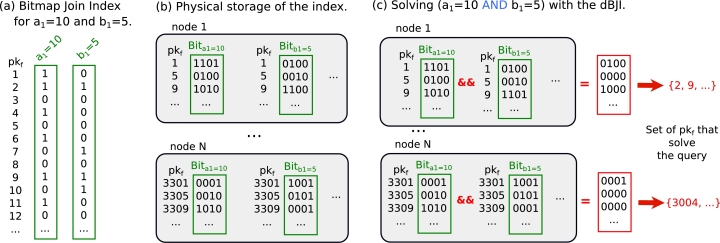
A representation of our distributed Bitmap Join Index (dBJI). **(a):** Example of instance of a bitmap index considering the same tables in Fig. 1(a). **(b):** Physical storage of the dBJI. **(c):** Example of application of the dBJI to solve an AND operation.

The Bitmap Join Index is composed of bitmap arrays that represent the occurrence of attribute values from dimension tables in the tuples of the fact table [20]. Star Join predicates can be solved by using bitwise logical operators on the bitmap indices, avoiding actual joins between fact tables and dimensions. Specifically, a Bitmap Join Index for an attribute *α* from the dimension table *D* is a set of bitmap arrays for every distinct value of *α*. For every value *x* of the attribute *α*, each bitmap $Bit_{\alpha =x}$ contains one bit for each tuple in the fact table, indexed by its primary key $pk_{f}$. Each of these bits represents the occurrence (1) or not (0) of the value *x* in the corresponding tuple of the fact table. We show in Fig. 3(a) examples for two attribute values, $a_{1}=10$ and $b_{1}=5$. Thus, for instance, if the *j*-th bit of the bitmap $Bit_{\alpha =x}$ is 1 (0), that means that the tuple on the fact table with $pk_{f}=j$ is (is not) associated with α=x. It is now evident that a predicate $\alpha_{1}=x_{1}⊗\alpha_{2}=x_{2}$, with ⊗ being a logical operator, can then be solved by evaluating the $Bit_{\alpha_{1}=x_{1}}⊗Bit_{\alpha_{2}=x_{2}}$ For instance, to find the tuples in the fact table under the condition $a_{1}=10\text{ AND }b_{1}=5$, the bitwise logical operator AND can be applied directly to the bitmaps $Bit_{a_{1}=10}$ and $Bit_{b_{1}=5}$. Thus, only tuples 2 and 9 from the fact table should be retrieved via random access.

The Bitmap Join Index has been proven a competitive solution, even when the number of indexed dimensions is large [29]. Although the cardinality of the dimension attributes is generally assumed to be small, a limitation of Bitmap Join Indices is handling attributes with high cardinality. These problems can be attenuated by optimization techniques, such as binning [30], [31], compression [32], [33], and coding [34]. As a result, the Bitmap Join Index is largely used to solve Star Joins queries in decision-making systems, especially data warehousing environments that store huge volumes of read-mostly data [31]. As expanded in the Section 4.2, in this paper we explore the benefits of Bitmap Join Indices in distributed systems.

### 2.2 Apache open-source software

**Hadoop MapReduce.** The Apache Hadoop MapReduce [6] (MapReduce for short) is an open-source distributed implementation of a generic programming model where Map and Reduce procedures manipulate key-value pairs [35]. In general, Map tasks filter and sort the data, and Reduce tasks summarize the results of the Map.

**Apache Spark.** The Apache Spark is a parallel and distributed framework based on in-memory computation [8] and on the Resilient Distributed Dataset (RDD) abstraction [36]. All operations on the RDDs are first mapped into a Directed Acyclic Graph (DAG) and then reorganized into sets of smaller tasks based on their mutual dependencies by the DAG scheduler.

**Apache HBase.** The Apache HBase is an open-source, NoSQL database designed to provide fast read and write operations to applications in Big Data [7]. Its primary goal is to store and manage huge tables on clusters based in cloud environments while leveraging the fault tolerance provided by the HDFS. Although HBase organizes data into tables in a similar fashion to relational databases, the data is denormalized, and there are no native joins.

To provide fast random access, HBase stores its tables in a custom format called HFile. Because the HDFS was designed for batch processing, datasets are usually split into large blocks (the standard block size is 64MB). These blocks are usually read in sequence. HBase uses the HFile format to build a second layer of smaller data blocks over the HDFS, with a standard block size of 64KB. Indices can be constructed on this second layer [37]: when a row key is requested, indices redirect the read/write operation to the location of the block where such row is stored. However, HBase reads the entire data block defined by the HFile format and then performs a sequential search to find that particular row key. Therefore, an HFile format with large block size adds up to the overhead during the reading operation and sequential search, while smaller block sizes require index data structures that are larger and more complex.

## 3 Related work

### 3.1 Joins in Hadoop

Because joining multiple tables is a common operation in many database applications and especially challenging when the data volume scales up, many of the systems in the Apache family support joins. For instance, Apache Hive [38] is a data warehouse solution built on top of Hadoop that performs join queries by processing a cascade of joins between each pair of tables (also known as cascade join or reduce-side join). When possible, Hive may also load small tables into the main memory and then compute the joins (also known as broadcast join). Both of these techniques consist of the common solutions to solve joins, and we will test the performance of these approaches by using the following terminology (see Table 1): *MR-Cascade* refers to a cascade join implemented in MapReduce; and *MR-Broadcast*, to the broadcast join. Blanas et al. [27] extensively studied the performance of these approaches, and other small variants, to provide a survey with guidelines to aid the selection of approaches when employing joins in MapReduce. For completeness, we explored their counterparts in Spark [24], the *SP-Cascade* approach for cascade join, and *SP-Broadcast* for the broadcast join.

**Table 1:** List of the approaches outlined (Section 3) and used in the performance evaluation (Section 5). The approaches proposed in this paper are highlighted in bold with gray background. The second and third columns distinguish the access method used by each approach (random access *vs.* full scan). The fourth and fifth columns identify optimization techniques, if any, as described in the main text (Section 3).

MapReduce algorithms	Random access	Full scan	Optimization	
			Filter	Broadcast
***MR-Bitmap***	✓			
*MR-Cascade*		✓		
*MR-MapKey*		✓		
*MR-Hierarchized*		✓		
*MR-Broadcast*		✓		✓
*MR-Bloom-ScatterGather*		✓	✓	
*MR-Bloom-MapKey*		✓	✓	
*MR-Bloom-Cascade*		✓	✓	
*MR-Bitmap-Filter*		✓	✓	



Spark algorithms	Random access	Full scan	Optimization	
			Filter	Broadcast
***SP-Bitmap***	✓			
***SP-Broadcast-Bitmap***	✓			✓
*SP-Broadcast*		✓		✓
*SP-Bloom-Cascade*		✓	✓	
*SP-Cascade*		✓		

### 3.2 Star Joins in Hadoop

Because in most applications the fact table is considerably large, the application of techniques that optimize how the data is handled is critical to process Star Joins efficiently. To avoid multiple MapReduce cycles, Afrati et al. [21] proposed a map-key approach (hereafter, *MR-MapKey*) that computes Star Joins in a single job by replicating and mapping data from dimension tables. Tao et al. [39] extended the *MR-MapKey* approach to perform hierarchical joins by adding more jobs. We refer to this extension as *MR-Hierarchized*. Although the *MR-MapKey* and *MR-Hierarchized* approaches avoid multiple jobs, there is significant replication of data among subsets of Reducers.

Another common strategy to accelerate the processing of Star Joins is the use of filters to prune the fact table before joining with the dimensions. For instance, one very simple improvement on top of the *MR-Cascade* is to use Bloom filters [40] to prune the fact table and perform the join with the dimensions, thus avoiding propagation of unnecessary data. We implemented this method in this paper, which is referred to as *MR-Bloom-Cascade*. Because *MR-Bloom-Cascade* requires as many jobs as dimension tables, Han et al. [22] proposed an approach (hereafter, *MR-Bloom-ScatterGather*) that only requires three jobs: (1) application of Bloom Filters, (2) join between each vertical partition of the fact table with corresponding dimensions and (3) merge of all vertical partitions. Zhang et al. [23] extended the *MR-MapKey* approach by including an additional job to build Bloom filters. We refer to this extension as *MR-Bloom-MapKey*. However, although these strategies proved the use of Bloom filters improved their performance, filtering requires one extra job. In analogy with *MR-Bloom-ScatterGather*, Zhu et al. [41] used Join Bitmaps as filters to prune the fact table, thus referred to as *MR-Bitmap-Filter*. Although this bitmap data structure used in this approach is similar to that presented in this paper, it employs full scan to retrieve data from the HDFS. Thus, the *MR-Bitmap-Filter* approach uses the bitmap data structure as a filter rather than an index, introducing an additional overhead to low-selectivity queries due to the lack of random access. In this paper, we redefine the bitmap index in a distributed context and employ random access to leverage the advantages of an index in solving low selectivity queries.

Finally, in our previous paper [24], we proposed and studied in detail two Spark approaches that minimize disk spill and network communication. Namely, these approaches are (i) the *SP-Bloom-Cascade*, which uses Bloom filters to prune the fact table and then performs the joins; and (ii) the *SP-Broadcast*, which implements the broadcast join. While the *SP-Broadcast* presented the best performance overall when enough memory was available, the *SP-Bloom-Cascade* strategy was remarkably resilient to scenarios with scarce memory per node, probably because the Bloom filters require minimal storage.

### 3.3 Contrasting our contribution

We summarize in Table 1 all approaches introduced so far, and include the approaches proposed in the present paper. Solutions are discriminated according to their access method (second and third columns) and whether they use optimization techniques (fourth and fifth columns). The approaches presented in this paper (namely *SP-Bitmap*, *SP-Broadcast-Bitmap*, and *MR-Bitmap*) are the only ones that employ random access, and excel at queries with low selectivity (as shown in Section 5.3). Because the underlying methodology only assumes two independent layers (one Access Layer and one Processing Layer), our solution is framework agnostic and can be further extended beyond the MapReduce and Spark frameworks. That is, to the best of our knowledge, there is no related work that employs *random access* to process Star Joins for data residing on a HDFS. Therefore, our proposal is innovative in the sense that, in a system intended for a general purpose the methodologies displayed in Table 1 can be selected on demand to solve a broader spectrum of queries, including high and low selectivity (see Sections 4.1 and 4.5).

## 4 Proposal

We introduce the following solutions to the fundamental challenges listed in Section 1:

1. We propose a strategy that operates on top of an architecture composed of a Processing Layer and an Access Layer that provides both full scan and random access (Section 4.1).
2. By employing an Access Layer able to perform random access, we propose a distributed Bitmap Join Index (dBJI) that leverages the parallelism provided by the Processing Layer to solve Star Joins (Section 4.2).
3. We present a scalable distributed algorithm for constructing dBJI (Section 4.3).
4. We propose an algorithm that solves low-selectivity join predicates using the dBJI (Section 4.4).
5. We instantiate the Access Layer with HBase, and the Processing Layer with either Spark or MapReduce (Section 4.5).

### 4.1 Combining processing and access layers

We propose the use of an architecture based on an Access Layer and a Processing Layer (Fig. 1c). Depending on the specific query, the Access Layer is responsible to employ either random access or full scan, and coordinate with the Processing Layer. This is important to separate concerns and not depend on technologies such as Spark to deliver random access. How the selection between random access and full scan is made should be addressed elsewhere. In fact, this strategy is similar to how relational databases solve queries by using query optimizers [42], [43]. Depending on which strategy is chosen, this system will behave in a slightly different way, as described below.

**Full scan.** The overall strategy is to request from the Access Layer a full scan of the fact table and the dimensions involved in the given query, and then the Processing Layer computes the join in parallel. We reviewed in Section 3.2 the existing algorithms available for full scan.

**Random Access.** In order to take full advantage of the parallel frameworks, the distributed index is first loaded and computed by the Processing Layer and, then, a request for the resulting tuples is sent to the Access Layer. Once the Access Layer receives this request, the tuples are retrieved from the appropriate cluster nodes using a random access method. The index loaded by the Processing Layer is called a secondary index, and the index used by the Access Layer is called a primary index. This strategy is described in detail in the next section.

### 4.2 Distributed Bitmap Join Index (dBJI)

The distributed Bitmap Join Index (dBJI) is a set of bitmap arrays that are stored on a distributed system and is designed to leverage the advantages of parallel processing to solve Star Joins. In the construction of the dBJI, it is not assumed that indices related to a partition of the tables are located on the same node (i.e., no assumption of collocation). For every value *x* of an attribute *α*, the bitmap array $Bit_{\alpha =x}^{P}$ contains one bit for each tuple in the fact table that is indexed by the primary keys $pk_{f}\in P$. The set *P* represents a subset of the primary keys of the fact table, and is used to split the index into many nodes. Let us assume that the sequence $P_{1},P_{2},\ldots ,P_{N_{P}}$ represents a partition of the complete set P of all primary keys that compose the fact table, with $N_{P}$ being the number of partitions. As in the original bitmap join index, each of these bits represents the occurrence (1) or not (0) of the value *x* in the corresponding tuple. Thus, the set of $pk_{f}$ that solve a simple predicate such as α=x is given by the list of all bits set to 1 in $Bit_{\alpha =x}^{P_{j}}$ in all partitions,

(1) $$S_{Q}=\overset{N_{P}}{\underset{j=1}{⋃}}\;\{\;pk_{f}\in P_{j}\;:\;Bit_{\alpha =x}^{P_{j}}[pk_{f}]=1\;\},$$

where $Bit_{\alpha =c}^{P_{j}}[pk_{f}]$ is the bit associated with $pk_{f}\in P_{j}$.

**Distributing the index.** To fully exploit parallelism and to balance the workload in the cluster, partitions are uniformly distributed across the nodes (Fig. 3b). Metadata containing information about the location of each partition is stored in the namenode (master). To optimize the loading time of the index files, the bitmap arrays $Bit_{\alpha =x}^{P_{j}}$ are stored in a different file for each attribute *α* and value *x* of that attribute. Without loss of generality, let us assume that the primary keys are the integers $1,2,3,\ldots ,N_{t}$, i.e., $pk_{f}\in Z\cap [1,N_{t}]$, with $N_{t}$ being the number of tuples in the database. Within partitions and for each attribute and attribute value, the bitmap arrays are organized into blocks of size $b_{s}$ that consists of: (i) the primary key $pk_{f}$ of the first tuple indexed by that block; and (ii) a sequence of $b_{s}$ bits that represent the bitmap values associated with the tuples between the $pk_{f}$ and $pk_{f}+b_{s}-1$. Fig. 3b shows an example of bitmap partitions with block size $b_{s}=4$ tuples.

**Solving join predicates.** Because the Star Joins often involve predicates with two or more distinct attributes or ranges of attributes, joins may require the use of multiple bitmap arrays associated with the same partition. Consider a query Q consisting of *m* predicates $p_{k}$ composed as $p_{1}{⊗}_{1}p_{2}{⊗}_{2}\ldots {⊗}_{m-1}p_{m}$, where ${⊗}_{k}$ is the logical operator linking predicates $p_{k}$ and $p_{k+1}$. Although extending this formalism to a wider range of predicates is straightforward (as mentioned in Section 2.1), our goal is to solve predicates $p_{k}\equiv \alpha_{k}=x_{k}$ involving an attribute $\alpha_{k}$ and one of its possible values $x_{k}$. To solve this chain of predicates, each node will compute a partial solution $Bit_{Q}^{P_{j}}$ as follows:

(2) $$Bit_{Q}^{P_{j}}=Bit_{\alpha_{1}=x_{1}}^{P_{j}}{⊗}_{1}Bit_{\alpha_{2}=x_{2}}^{P_{j}}{⊗}_{2}\ldots {⊗}_{m-1}Bit_{\alpha_{m}=x_{m}}^{P_{j}}.$$

Finally, the set of all required primary keys,

(3) $$S_{Q}=\overset{N_{P}}{\underset{j=1}{⋃}}\{\;pk_{f}\in P_{j}\;:\;Bit_{Q}^{P_{j}}[pk_{f}]=1\;\},$$

is aggregated by the master node and sent to the Access Layer. If a precedence order is required, the same precedence should be applied on the chain of operations with bitmap arrays. For example, the predicate $(a_{1}=10\text{ AND }b_{1}=5)\text{ OR }b_{2}=10$, similar to the predicate in Fig. 3c, is solved by performing

(4) $$Bit_{Q}^{P_{j}}=(Bit_{a_{1}=10}^{P_{j}}\wedge Bit_{b_{1}=5}^{P_{j}})\vee Bit_{b_{2}=10}^{P_{j}}$$

for $j=1,2,\ldots N_{P}$, where ∧ is the bitwise logical AND operator and ∨, the OR operator.

**Complexity analysis.** Because the cardinality $\left|S_{Q}\right|$ is proportional to the selectivity of the query Q, low-selectivity queries have low memory demand. Additionally, because Star Joins are solved by employing bitwise operations on bitmap arrays $Bit_{\alpha =x}^{P_{j}}$ that share the same partition, the index structures with the same partition should ideally be stored on the same node. If not, then the index needs to be appropriately transmitted across the cluster, creating additional delays on the query processing. Finally, because computing $S_{Q}$ involves computing a cascade of predicates and an aggregation, it is important to study its complexity:

Theorem 1 *For a given query Q consisting of m predicates* $p_{k}$ *composed as* $p_{1}{⊗}_{1}p_{2}{⊗}_{2}\ldots {⊗}_{m-1}p_{m}$*, where* ${⊗}_{k}$ *are logical operators, computing* $S_{Q}$ *has time complexity* $O((m+1)N_{t})$*, where* $N_{t}$ *is the number of tuples in the fact table.*

Proof If m=0, then there is only one predicate $p_{k}\equiv \alpha =x$ and $S_{Q}$ is computed by the aggregation in Equation (3) for all partitions $Bit_{\alpha =x}^{P_{j}}$. Because that requires scanning all $N_{t}$ elements of the bitmap structure, the complexity in this case is $O(N_{t})$. If m>0, then for each pair of predicates $p_{j}{⊗}_{j}p_{j+1}$ with j=1,…,m, first the bitwise logical operation between the bitmaps associated with predicates $p_{j}$ and $p_{j+1}$ are computed (as in Equation (2)). Given a number $N_{P}$ of partitions with same size, each bitwise operation has time complexity $O(N_{t}/N_{P})=O(N_{t})$. After performing all operations, the final aggregation of all primary keys is performed, which results in a total complexity of $O((m+1)N_{t})$. □

### 4.3 Constructing the dBJI

Because the size of the index structure scales with the number of tuples in the fact table, we propose in Algorithm 1 a distributed algorithm to construct and store the dBJI on a distributed file system. Our algorithm controls how the partitions are distributed in the cluster, the size of each partition and the block size $b_{s}$. It receives as input the fact (*F*) and dimension (*D*) tables, the attribute (*α*) and value (*x*) being indexed, the number of partitions ($N_{P}$) and the block size ($b_{s}$). Algorithm 1 starts by collecting the primary keys from the dimension table and stores the keys that correspond to the indexed value (i.e., α=x) into a hash map (lines 1-6). Then, the fact table is split into $N_{P}$ horizontal partitions (line 7). Each partition is processed and generates a corresponding bitmap partition (line 8-29). Within each partition, a new bitmap block is created to incorporate the next $b_{s}$ tuples (line 11). The primary key of the first tuple is stored at the beginning of the blocks (line 12). Next, the boolean values indicating whether the tuple *t* has the indexed value or not generate bitmap blocks (lines 14-25). Finally, the algorithm outputs the dBJI for α=x. We provide a MapReduce implementation of Algorithm 1 on Appendix A. The implementation is also available at GitHub [28].

**Algorithm 1:**
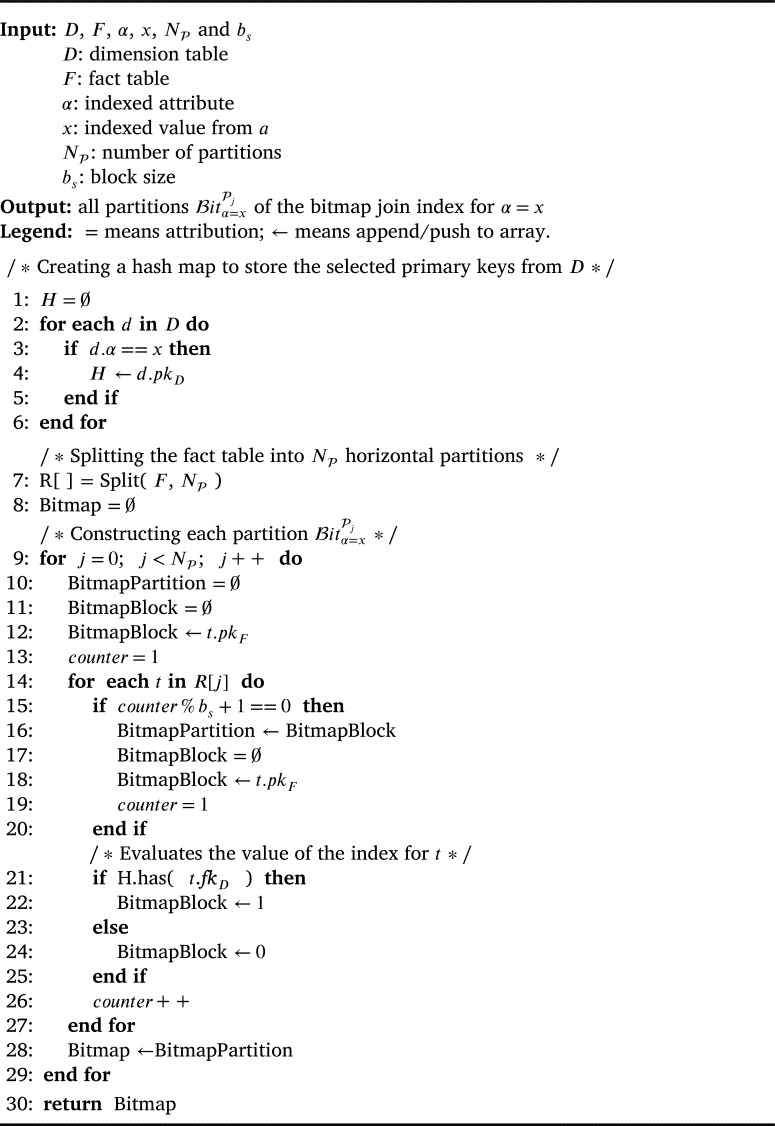
Constructing the dBJI.

Theorem 2 Algorithm 1 *has time complexity* $O(N_{t})$*, where* $N_{t}$ *is the number of tuples in the fact table.*

Proof For a given dimension *D* with $N_{d}$ entries, the complexity to compute lines 1-6 is $O(N_{d})$. Between lines 9-29, the two nested loops scan the fact table a single time. Thus, the complexity becomes $O(N_{d})+O(N_{t})$. Because in star schemas $N_{d}\ll N_{t}$, the complexity is $O(N_{t})$ regardless of the number $N_{P}$ of partitions or the block size $b_{s}$. □

### 4.4 Processing Star Joins with the dBJI

The Star Join processing pipeline proposed in this paper is divided in two phases. First, for a query Q the Processing Layer computes $Bit_{Q}^{P_{j}}$ in each node using the distributed Bitmap Join Indices (dBJI). The corresponding bitmap partitions are then combined into a set $S_{Q}$ of primary keys. As discussed in Section 4.2, because we store corresponding partitions in the same nodes, this procedure is executed locally. The second phase consists of sending $S_{Q}$ to the Access Layer to execute random access based on its primary indexing schema. Thus, only the required tuples from the fact table are retrieved from the distributed filesystem and joined to the dimension tables.

**dBJI as a secondary index with loose binding.** To successfully employ this strategy with existing distributed systems, the dBJI connects to a primary index used in the distributed random access performed by the Access Layer. The primary index is a map between a primary key $pk_{j}$ and an address $\text{PI}(pk_{j})$ in the distributed filesystem (specifying cluster node and disk address). Thus, for a set of primary keys $S_{Q}$ that solve a query Q, a function DistributedRandomAccess(⋅) interfaces with the Access Layer to employ random access to retrieve records from addresses $\text{PI}(pk_{j})$, $\forall pk_{j}\in S_{Q}$. Although this two-level structure adds a small overhead, it does not change the complexity of the Star Join and can be minimized if performed in bulk.

**Figure 4:**
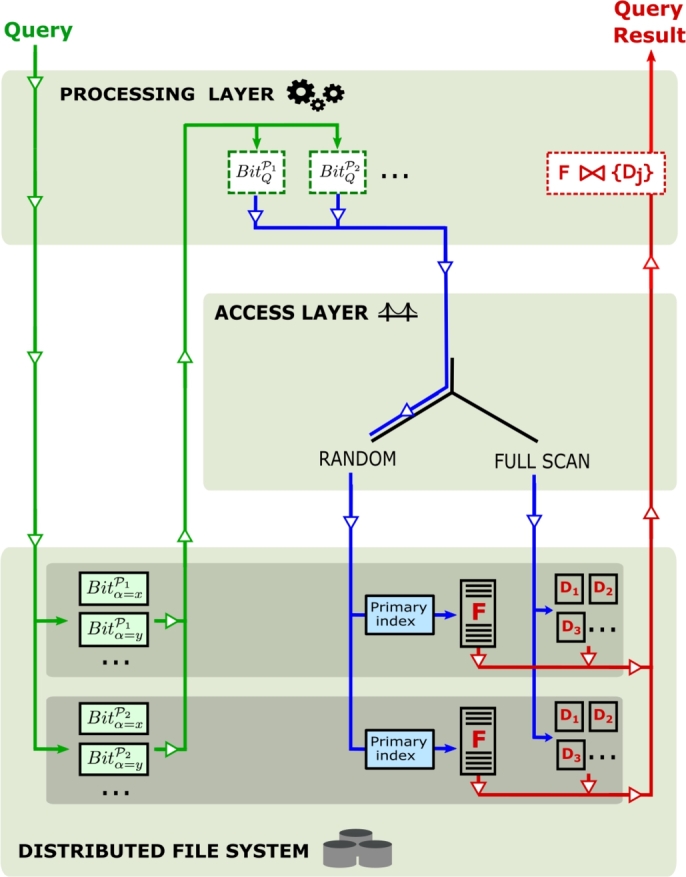
Workflow of our solution on a Hadoop-based instance of the architecture and distributed Bitmap Join Index (dBJI).

**Processing algorithm.** Algorithm 2 computes the Star Join using random access, as depicted by the workflow in Fig. 4. The algorithm receives as input a Star Join query *Q*, a fact table *F*, a set of dimensions $\left\{D_{j}\right\}$, and a set of dBJIs {Bit}. Lastly, the argument JoinMethod(⋅) of Algorithm 2 can be any join algorithm (e.g. cascade join or a broadcast join). The algorithm starts by the Processing Layer loading the necessary bitmap structures and construct S=Q∪jBitQPj on line 1 (green lines in Fig. 4). In line 2, this list is passed through DistributedRandomAccess(⋅) to the Access Layer to employ the primary index and retrieve the necessary tuples from the fact table (blue lines). Then, the dimension tables required in the query *Q* are loaded via full scan (blue lines below the Full Scan sign), filtered, and joined with the fact table in lines 3-6 (red lines). If necessary, the result can be grouped and/or sorted between lines 6 and 7, depending on the clauses present in the query *Q*.

**Algorithm 2:**
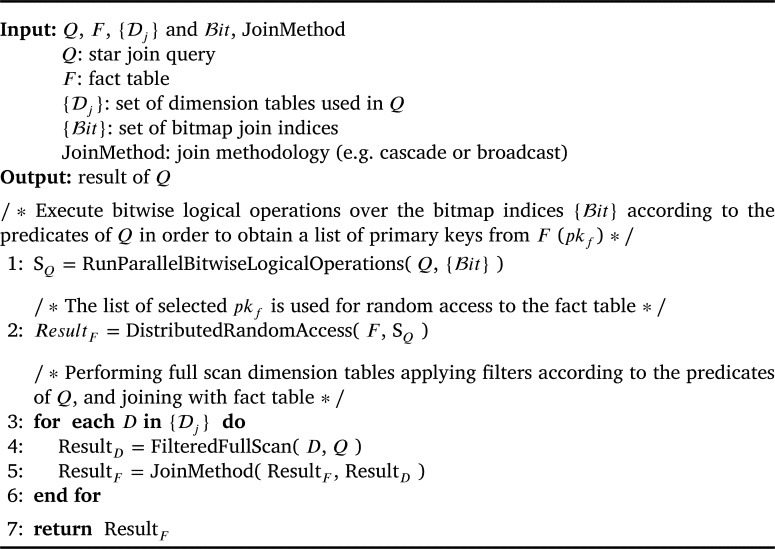
Processing Star Join Queries with the dBJI.

**Complexity.** Because of the complexity in evaluating Q from Theorem 1, Algorithm 2 processes Star Joins in linear time. Although the specific methodology depends on the specific implementation, the distributed random access performed in line 2 retrieves the list in Q one by one, i.e., it runs in linear time. Lines 3 through 6 in Algorithm 2 retrieve the dimensions involved and performs a join operation.

### 4.5 Instance based on Hadoop systems

Both the architecture presented in Section 4.1 and the dBJI can be implemented in a distributed system based on Hadoop-related software. The most natural candidate for the distributed filesystem is the HDFS. The Processing Layer can be realized by either MapReduce or Spark frameworks to deliver massive parallel computations. Finally, HBase is a good candidate for Access Layer for its trade-off between full scan and random access and the fact that HFile are suitable for star schemas. The connection between the dBJI and HBase's primary index is performed through its API function BulkGet(⋅). There are two important low-level features specific to HBase that may affect the performance of our system: (i) how HFile blocks are read and (ii) their size. First, although HBase's BulkGet(⋅) can use a primary index to locate HFile blocks associated with a set of primary keys, it performs a sequential search within HFile blocks. Second, the block size used by HBase HFile (64KB) is larger than the common size employed by most relational systems (PostgreSQL's default page size is 8KB). This difference in block size influences the performance of random access due to additional read/write. We investigated the effect of the block size empirically in section 5.6. We provide two implementations of Algorithm 2 in Appendices B and C (also in GitHub [28]). This setup and algorithms are used in Section 5 to validate the performance of our proposal.

## 5 Performance evaluation

In this section, we evaluate the performance of the proposed strategies using the distributed Bitmap Join Index (dBJI) and compare it to those of previously published algorithms. To perform both random access and full scan, we use the architecture in Fig. 2 and the instantiation proposed in Section 4.5. We explored both MapReduce and Spark as candidate Processing Layers and HBase as an Access Layer. All implementations used in this section are available on GitHub [28].

### 5.1 Experimental setup

**Cluster.** We set up a cluster in Microsoft Azure with 1 master and 20 slave D3v2 instances. Each instance had four 2.4 GHz Intel Xeon E5-2673, 14GB of memory, and a hard disk of 1TB. We used Hadoop MapReduce 2.6.0 and Spark 1.4.1 as processing engines, YARN 2.6.0 as cluster manager, and HBase 1.1.2. It is worth noting that Apache Spark 2 became stable after we performed our experiments, and recent optimizations of Apache Spark 2 may reflect in further performance gains for our proposed strategy, which combines a distributed Bitmap Join Index (dBJI) and a two-layer architecture based on open-source frameworks to speed up Star Join queries with low selectivity.

**Dataset.** We used the Star Schema Benchmark (SSB) [44] to generate synthetic datasets to study the performance of Star Joins. The size of each dataset was controlled by the Scaling Factor (SF). Table 2 shows detailed information about the datasets used in our tests. Each table was stored into a single column family and partitioned across 600 HBase regions. We used the default size for HBase blocks (64KB). We abbreviated the column names and the family qualifiers to reduce the data volume because HBase replicates this information for every value.

**Table 2:** Characteristics of the datasets and bitmap indices used in the performance evaluations.

SF	Number of tuples (×10^8^)	Size (GB) of each dataset on disk	Tuples per Hbase region (×10^6^)	Size (MB) of each bitmap array	Number of partitions per bitmap array
100	0.6	326	1	71.9	100
200	1.2	655	2	143.4	200
300	1.8	982	3	214.6	300
400	2.4	1310	4	286.2	400

**Workload.** We used four Star Join queries with low selectivity from the SSB, namely Q2.3, Q3.3, Q3.4, and Q4.3. Table 3 details the queries used in our tests. The queries defined on the SSB approximately maintain their selectivity regardless of the SF. To test the impact of increasing query selectivity values on the computation time, we changed the predicate of the query Q4.3 and created queries Q4.4, Q4.5, and Q4.6, also defined in Table 3. All results represent the average over 5 runs, and bars represent the standard error. Queries from the SSB or similar to them are largely used to compare competing solutions to Star Joins in cloud environments [22], [24], [39], [41]. Although these queries generally encompass at most four Star Join operations, our proposal nicely scales for queries with more operations since its complexity varies linearly with the number of predicates, as stated in Theorem 1. To ensure that each replicate was not influenced by the cache, all nodes' memories were flushed between each execution.

**Table 3:** Predicate and approximate selectivity of all queries used in our performance evaluations.

Query	Predicates	Selectivity (%)
Q2.3	*p*_*brand* = ‘MFGR#2221’ and *s*_*region* = ‘EUROPE’	≈0.020
Q3.3	(*c*_*city* = ‘UNITED KI1’ or *c*_*city* = ‘UNITED KI5’) and (*s*_*city* = ‘UNITED KI1’ or *s*_*city* = ‘UNITED KI5’) and *d*_*year* > =1992 and *d*_*year* < =1997	≈0.0059
Q3.4	(*c*_*city* = ‘UNITED KI1’ or *c*_*city* = ‘UNITED KI5’) and (*s*_*city* = ‘UNITED KI1’ or *s*_*city* = ‘UNITED KI5’) and *d*_*yearmonth* = ‘Dec1997’	≈0.000083
Q4.3	*c*_*region* = ‘AMERICA’ and *s*_*nation* = ‘UNITED STATES’ and (*d*_*year* = 1997 or *d*_*year* = 1998) and *p*_*category* = ‘MFGR#14’	≈0.0077
Q4.4	*c*_*region* = ‘AMERICA’ and *p*_*category* = ‘MFGR#14’ and *d*_*year* = 1998	≈0.071
Q4.5	*c*_*region* = ‘AMERICA’ and *p*_*category* = ‘MFGR#14’ and *d*_*year* > =1996 and *d*_*year* < =1998	≈0.31
Q4.6	*c*_*region* = ‘AMERICA’ and *p*_*category* = ‘MFGR#14’ and *d*_*year* > =1994 and *d*_*year* < =1998	≈0.56

**Distributed Bitmap Join Indices (dBJI).** The indices dBJI necessary to solve the predicates in Table 3 were constructed following Algorithm 1. Each bitmap array was split into 100 partitions and with the block size corresponding to 3 million tuples (Table 2). The indices were stored as sequence files in HDFS and were distributed across the 20 slave nodes. We did not assume collocation between partitions of the dBJI and the fact table. Further, because our primary goal is to evaluate the solution of Star Joins with random access in distributed systems, we did not apply any of the optimization techniques listed in Section 2.1 (i.e., binning, compression, and coding). For instance, binning could be used to improve the structure when indexing attributes that can assume a large range of values or numeric values that are not discrete.

**Tested algorithms.** We compared the performance of our proposed algorithms *SP-Bitmap*, *SP-Broadcast-Bitmap*, and *MR-Bitmap* against 11 different approaches based on full scan, as summarized in Table 1. This table groups the strategies by the following criteria: (i) access method (random access *vs.* full scan); (ii) processing framework employed (MapReduce *vs.* Spark); and (iii) whether filters or broadcasting were used.

### 5.2 Optimization of MapReduce parameters

To ensure that the performance of MapReduce strategies can be compared to that of Spark strategies, we optimized the two parameters that are influential in performance tests: the *number of reducers* and the *slow start ratio*. The number of reducers defines how many reducers are concurrently instantiated in each Map/Reduce cycle. In some strategies, the number of reducers should present a strong impact because it induces an increased amount of replicated data that is transferred during the join operation. The slow start ratio defines the number of map tasks that must be completed before scheduling reduce tasks for the same job. By default, this ratio is 0.05. For the tests reported in this section, we used a dataset with SF=100 and investigated queries Q2.3, Q3.3, Q3.4, and Q4.3.

**Figure 5:**
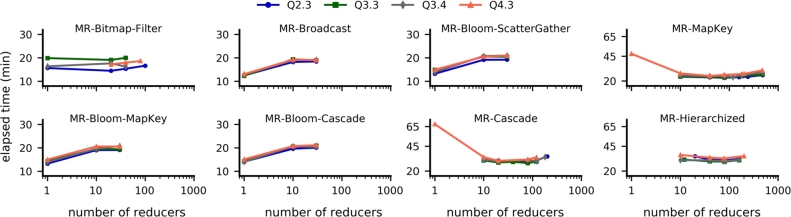
Performance of the MapReduce strategies based on full scan as a function of the number of reducers.

**Figure 6:**
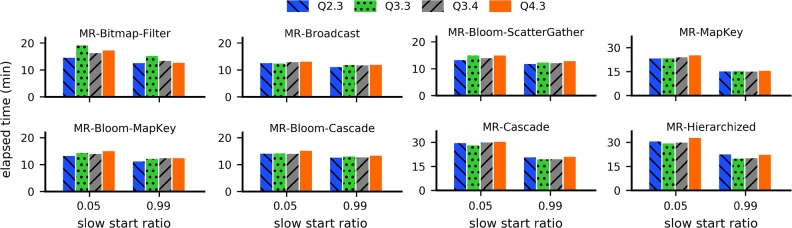
MapReduce strategies based on full scan showed better performance with a value of slow start ratio equal to 0.99.

All of the MapReduce strategies based on full scan presented a region of values of the number of reducers in which their performance was either optimal or very close to optimal, regardless of the query (Fig. 5). In particular, the optimal number of reducers was higher for strategies that do not apply filtering optimizations (e.g., *MR-MapKey* and *MR-Cascade*). This is probably because the total workload, including data shuffling and processing, was balanced across all the available reducers. Approaches based on filtering techniques were optimized with very few reducers (e.g., *MR-Bloom-MapKey* and *MR-Bloom-ScatterGather*). The only exception was the *MR-Bitmap-Filter*: its performance remained stable for a number of reducers below 100. Furthermore, all full scan MapReduce strategies showed an improvement in performance when the slow start ratio was set to 0.99, which is a higher value than the default (Fig. 6). The improvement in performance ranged from 9.6% to 49.9%.

**Figure 7:**
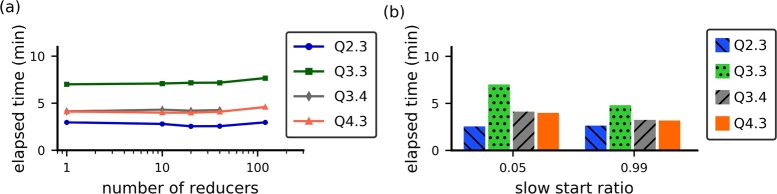
Performance of our proposed *MR-Bitmap* as a function of (a) the number of reducers and (b) the slow start ratio.

Finally, the *MR-Bitmap* strategy, which we propose in this paper, showed optimal performance with a low number of reducers and the same high value for the slow start ratio (Fig. 7). For a number of reducers smaller than 100, the performance of our approach remained mostly constant (Fig. 7a). Regarding the slow start ratio (Fig. 7b), the computation time either remained the same (query Q2.3) or improved by a factor between 24% and 36%.

Based on the results described in this section, in each strategy we set the number of reducers to the value corresponding to their best elapsed time, according to Figs. 5 and 7(a). We also set the slow start ratio of all algorithms to 0.99.

### 5.3 Performance across different approaches

**Figure 8:**
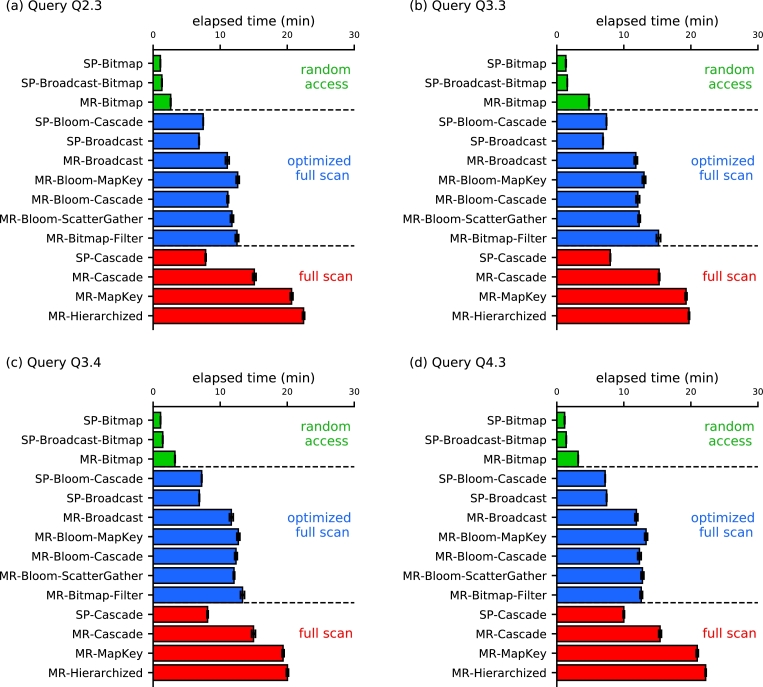
The proposed strategies *SP-Bitmap*, *SP-Broadcast-Bitmap* and *MR-Bitmap* (green bars) outperformed all full scan strategies for all queries with low selectivity. Strategy names follow Table 1. Red and blue bars refer to full scan approaches. Optimized approaches (blue bars) refer to the use of filters and broadcast (indicated at the last column of Table 1).

Especially in queries that have low selectivity, our strategies, based on the dBJI, presented the best performance regardless of the framework used for their implementation (compare green bars with red and blue in Fig. 8). For the tests reported in this section, we used a dataset with SF=100 and investigated queries Q2.3, Q3.3, Q3.4, and Q4.3. Regarding the MapReduce framework, our strategy *MR-Bitmap* dropped the computation time by a factor between 39.7% and 88.3%. In the Spark framework, the performance increase of our strategies *SP-Bitmap* and *SP-Broadcast-Bitmap* varied from 77.3% up to 88.3%. These results demonstrate the advantage of associating *random access* with the use of our dBJI to process low selectivity Star Join queries. Moreover, although Spark implementations tend to outperform those in the MapReduce framework, our *MR-Bitmap* algorithm outperformed all Spark strategies based on full scan by a factor from 30.3% up to 68.2%. Therefore, the application of the appropriate access method may have a stronger influence on query performance than that of the choice of framework.

Because *MR-Bitmap-Filter* employs a Bitmap Join structure to filter the fact tables, the fact that it significantly underperforms our method *MR-Bitmap* agrees with our overarching hypothesis that the proper use of random access is likely to improve the performance of Star Joins substantially. Moreover, the *MR-Bitmap-Filter* was slightly slower than approaches that use Bloom filters with full scan (*MR-Bloom-ScatterGather*, *MR-Bloom-MapKey*, and *MR-Bloom-Cascade*). Despite this fundamental difference, both *MR-Bitmap-Filter* and *MR-Bitmap* were the only two that presented a constant performance for a number of reducers smaller than 100 (Figs. 7 and 5).

Comparing MapReduce solutions based on full scan, the use of filters significantly reduced the computation time (compare blue and red bars in Fig. 8). For the MapReduce strategies, the use of optimizations improved the elapsed time from 0.7% up to 50.7%. For the Spark strategies, the optimized full scan strategies showed an improvement in the performance ranging from 7.0% to 37.9%. Considering all full scan strategies, the Spark algorithms outperformed those in MapReduce, reducing the computation time by a factor of between 15.8% to 69.5%.

For the remainder of the performance tests, we will only use the best two approaches based on full scan to compare with our proposed methods. Based on the results presented in this section, the two MapReduce strategies with computation times closest to that of our *MR-Bitmap* were the full scan strategies *MR-Broadcast* and *MR-Bloom-MapKey*. The Spark strategies with the computation times closest to that of our *SP-Bitmap* and *SP-Broadcast-Bitmap* were the full scan *SP-Bloom-Cascade* and *SP-Broadcast*.

### 5.4 Effect of query selectivity

**Figure 9:**
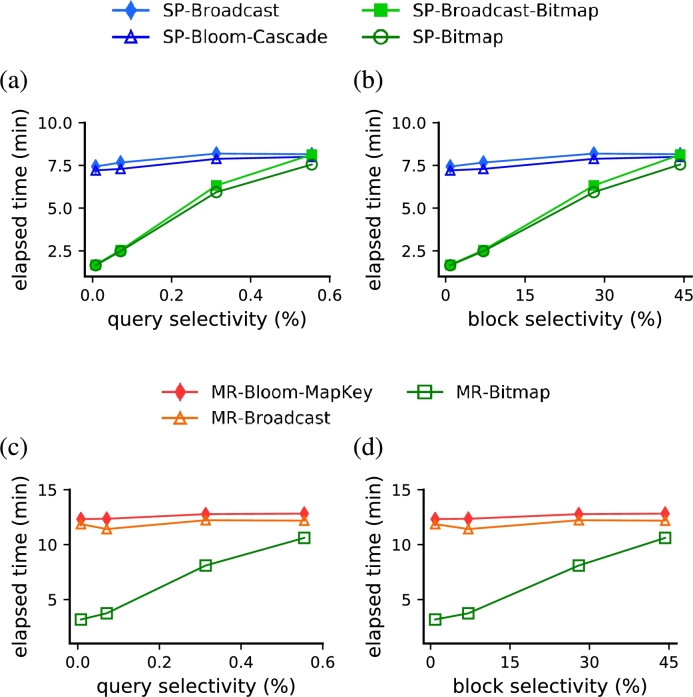
The proposed strategies based on random access (green markers), both for Spark and MapReduce, outperformed the two fastest full scan strategies (blue markers) when the query selectivity was smaller than 0.6%. (a,c) Performance results as a function of query selectivity. (b,d) Performance results as a function of block selectivity.

Our strategies based on random access outperformed competitor strategies based on full scan when the query selectivity was below 0.6% (Figs. 9a,c). In these experiments, for each framework (Spark and MapReduce) we compare our algorithms with the two full scan runner-up approaches, as indicated at the end of Section 5.3. We fixed the dataset with SF=100 and used queries Q4.3, Q4.4, Q4.5 and Q4.6, which have increasing query selectivity values (Table 3). In the Spark framework, both *SP-Broadcast-Bitmap* and *SP-Bitmap* outperformed *SP-Bloom-Cascade* and *SP-Broadcast* that use full scan for all query selectivities under 0.6% (Fig. 9a). When the query selectivity was below 0.2%, using the dBJI resulted in a performance gain ranging from 62% to 78% with respect to the full-scan strategies. Similarly, the *MR-Bitmap* also outperformed *MR-Broadcast* and *MR-Bloom-MapKey* in the same region of values for the query selectivity (Fig. 9c). The best performance results of *MR-Bitmap* were also with query selectivities below 0.2%, providing a performance gain ranging from up 67% to 74%. When query selectivity was 0.56% (query Q4.6), *SP-Broadcast-Bitmap* trailed its full scan counterparts, *SP-Bloom-Cascade* and *SP-Broadcast*, by a factor of 1.6%. Additionally, *SP-Bitmap* and *MR-Bitmap* still outperformed their respective Spark and MapReduce counterparts but by a small margin.

### 5.5 Effect of block selectivity

In analogy to standard relational databases, solutions based on indices tend to outperform other methodologies in a broader range of selectivity values (in some cases, at least up to 5% [18]). The discrepancy between the range of selectivity in which random access solutions are preferred can be explained by two independent factors: (i) because the dBJI is a secondary index with loose binding, there is an additional overhead in the communication with the primary index; and (ii) because HBase performs sequential searches within the HFile blocks, while standard relational databases use offsets to locate a record within a data block. Hereafter, we define the fraction of blocks retrieved to solving a query as its *block selectivity*. Indeed, using the same queries and SF as in Section 5.4, the block selectivity in which our strategies based on indices outperformed those based on full scan ranged up to 45% (Fig. 9b,d). This observation means that 45% of the total of HFile blocks were accessed.

**Figure 10:**
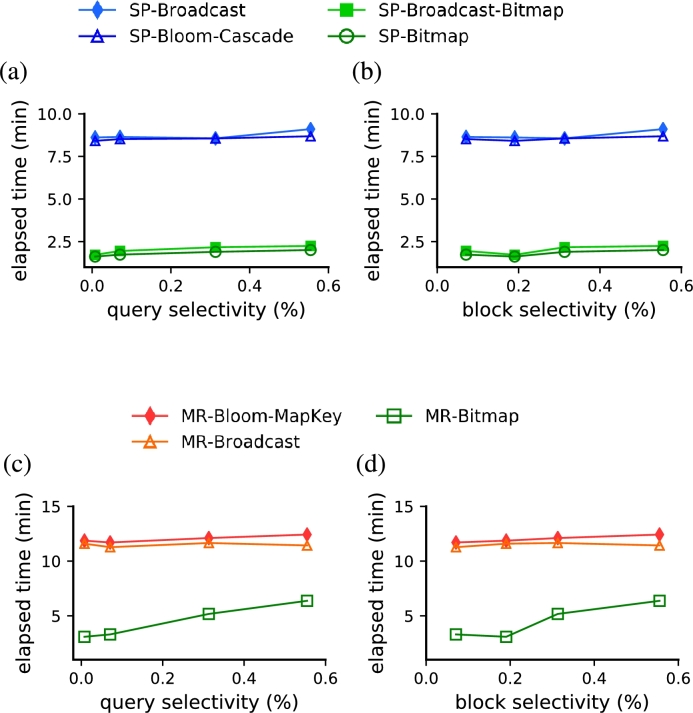
The performance of our strategies based on random access (green markers) outperformed the two fastest full scan strategies (blue markers) on a broader range of selectivity values when the database was sorted. (a,c) Performance results as a function of query selectivity. (b,d) Performance results as a function of block selectivity.

Sorting the dataset by some of the query predicates reduced the block selectivity dramatically, consequently decreasing the computation time of our strategies based on random access (Fig. 10). We sorted the fact table according to the predicates of query Q4.4 (i.e., d_year, c_region and p_category). With the database sorted, the block selectivity (Figs. 10b,d) decreased substantially (from 45% to 0.6%) and was comparable to the query selectivity (Figs. 10a,c). Furthermore, comparing results from Figs. 10(a,c) and 9(a,c) shows that our strategies based on random access became robust to a larger range of values of both query and block selectivities. On average, *SP-Bitmap* and *SP-Broadcast-Bitmap* were 78% faster than *SP-Bloom-Cascade* and *SP-Broadcast* (Fig. 10a,b). *MR-Bitmap* also outperformed the best MapReduce strategies based in full scan by a factor between 44% and 74% (Fig. 10c,d).

### 5.6 Effect of block size

**Figure 11:**
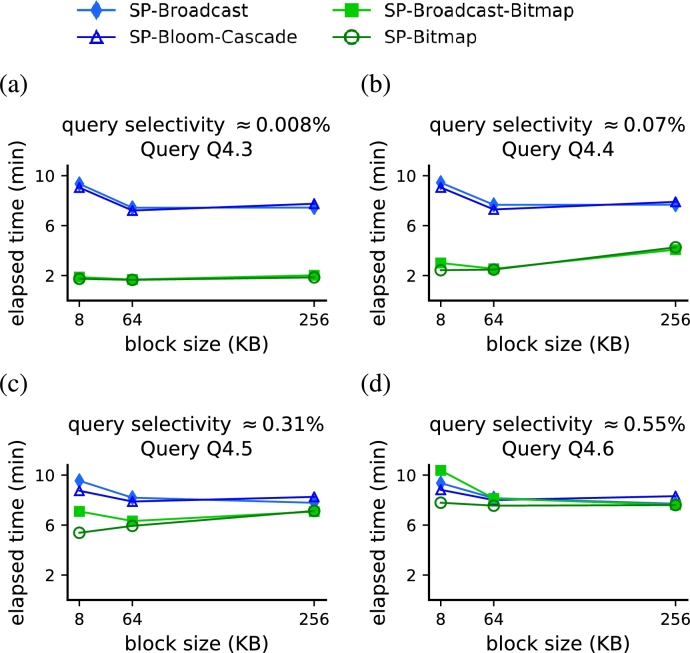
All strategies presented their best performances with a HFile block size of 64 KB, regardless of the query selectivity.

For methods based on random access, there exists a trade-off between a large block size (excessive readout) and a small block size (increased disk seeks and index complexity). Setting a block size in any of these extremes dropped the computation time of Star Joins by approximately 15% (Fig. 11). We report performance results with HFile block sizes of 8KB, 64KB and 256KB, using a dataset with SF=100 and queries Q4.3, Q4.4, Q4.5, and Q4.6. Regardless of the query selectivity, our algorithms *SP-Bitmap* and *SP-Broadcast-Bitmap* presented their best performances when the block size was 64KB. The performance of *SP-Broadcast-Bitmap* slightly dropped 14.6% when the block size was 8KB (compared to a 64KB block size). This is due to the increase in the main memory required to store block indices and hash maps. Furthermore, the performance of *SP-Broadcast-Bitmap* and *SP-Bitmap* dropped 16% when the block size was increased to 128KB (compared to a 64KB block size). This drop in performance was caused by the unnecessary amount of data read and increased sequential searches. For the smallest selectivity value (Fig. 11a), the performance gains of our algorithms remained remarkably constant. Interestingly, the full scan strategies also presented their best performances with a block size of 64KB, regardless of the query selectivity. Because once the database is deployed the block size cannot be changed, this result is important to show that, regardless of the access method (full scan or random access), all strategies perform the best at the same block size.

### 5.7 Effect of the data volume

**Figure 12:**
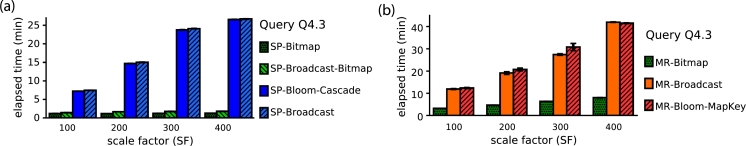
The performance of our proposed algorithms (green bars) presented a linear dependency with the scaling factor of the dataset.

Regarding the scalability of our approaches, the performance of *MR-Bitmap*, *SP-Bitmap* and *SP-Broadcast-Bitmap* remained linear with the Scaling Factor *SF* (Fig. 12). We report performance evaluations for query Q4.3, with a block size of 64KB and varying SF. The performance of our Spark solutions *SP-Bitmap* and *SP-Broadcast-Bitmap* remained remarkably constant with respect to *SF* (Fig. 12a). Comparing the two full scan runner-up approaches, as indicated at the end of Section 5.3, both *SP-Bitmap* and *SP-Broadcast-Bitmap* outperformed by a factor between 83% and 94%. In MapReduce, our strategy *MR-Bitmap* outperformed both *MR-Broadcast* and *MR-Bloom-MapKey* by a factor between 74% and 81% (Fig. 12b).

## 6 Conclusions

In this paper, we proposed a distributed Bitmap Join Index (dBJI) and an index-based distributed strategy to compute Star Joins that overcomes the lack of broad support to random access in available open-source distributed systems. Our solution is based on an architecture composed of two independent elements: a Processing Layer that performs massively distributed computation; and an Access Layer that delivers both full scan and random access on demand. The Access Layer serves as a middleware that supports random access between the HDFS and processing frameworks (Spark and MapReduce, in our tests). Among all eleven alternative solutions tested, three were based on Spark [24], and eight were based on the MapReduce framework [21], [22], [23], [27], [39], [41].

Our experiments showed that dBJI and an efficient processing algorithm outperform alternative full scan solutions for queries with low selectivity (Fig. 8). In our implementation, the dBJI used HBase's API to perform random access on HFiles and retrieve only the blocks that were pertinent to solve the query at hand. We noticed, however, that the HFile format slightly inflated the dataset, which had a small negative influence on the computation. The gain in computation time due to the dBJI, however, makes up for the additional delay due to the data inflation.

We also learned that a query optimizer should take into consideration, in addition to the estimated query selectivity, the proportion of blocks to be accessed, namely the block selectivity, when deciding between random access and full scan. Indeed, the strongest impact in the computation time was observed when the block selectivity was significantly reduced as a consequence of sorting the dataset (compare panels b and d from Figs. 9 and 10). Although this fact indicates that a large block size penalizes the computation time of the indexed strategies probably due to the repeated sequential searches within blocks, computation time is also hurt by a block size too small. Therefore, a distributed file system's block size should poise both of these opposing effects (as shown in Fig. 11) to deliver an optimal query performance. We also confirmed a pronounced increase in the performance of queries with predicates that involve an attribute sorted in the distributed file system (compare performance in Figs. 9 and 10). Thus, besides improving the performance, the range of block selectivity under which random access solutions were optimal became broader (Figs. 10). This indicates that the use of indices becomes more robust and is likely very favorable whenever predicates use sorted attributes.

As Star Joins are ubiquitous and expensive operations, our contribution consists of a general strategy to handle low-selectivity queries and mitigate the absence of native methods for random access in, for instance, the Hadoop software family. There is, of course, much ground to be covered in terms of construction, optimization, and use of indices to solve complex queries and analytical processes in the cloud. One strong candidate that offers native support to random access is the Apache Kudu [45], a distributed storage specialized in fast analytics. It provides an intermediary step between the fast capabilities provided by Apache Parquet [46] for full scans, and the nimble random access provided by HBase. In addition, Pilosa [47] is another open-source project that offers distributed bitmap indexing with the promise to accelerate queries of massive datasets. As the gap in support for random access hinders further development of such a promising research area, the concept behind combining a secondary index with Access and Processing Layers can be extended to implement other kinds of indices (e.g., B-Trees) and operations (e.g., drill-down). Furthermore, many studies may directly or indirectly benefit from using proper random access in distributed systems, such as [41], [48], and our paper paves the way for such applications with minimal tailoring and tinkering. We believe that our ideas may contribute to foster discussion and collaborative efforts to create novel tools that are also openly available to the community.

## Declarations

### Author contribution statement

C.D.A. Ciferri, J.J. Brito, T. Mosqueiro, R.R. Ciferri: Conceived and designed the experiments; Performed the experiments; Analyzed and interpreted the data; Contributed reagents, materials, analysis tools or data; Wrote the paper.

### Funding statement

J.J. Brito, T. Mosqueiro and C.D.A. Ciferri acknowledge Microsoft Azure Research Award MS-AZR-0036P. J.J. Brito acknowledges support from FAPESP grant 2012/13158-9. C.D.A. Ciferri acknowledges support from FAPESP grant 2018/22277-8. R.R. Ciferri acknowledges support from CNPq grant #311868/2015-0. This study was financed in part by the Coordenação de Aperfeiçoamento de Pessoal de Nível Superior – Brasil (CAPES) – Finance Code 001.

### Competing interest statement

The authors declare no conflict of interest.

### Additional information

Supplementary material related to this article can be found online at https://doi.org/10.1016/j.heliyon.2020.e03342.

No additional information is available for this paper.

## Supplementary material

Appendices Appendix A-C. Appendices
