# Supplementary material for: Random access with a distributed Bitmap Join Index for Star Joins
Source: Heliyon. 2020 Feb 17;6(2):e03342. doi: 10.1016/j.heliyon.2020.e03342 (PMC7030998; doi:10.1016/j.heliyon.2020.e03342)
Supplement: Appendices — Appendix A-C. [file mmc1.pdf]

Appendix A. Construction Algorithm of the distributed Bitmap Join Index (dBJI) with MapReduce

The MapReduce implementation of the bitmap index construction is detailed in Algorithm 3. The algorithm starts by loading the dimension table into the memory of each node at the Map Setup function. The fact table partitions are processed in the Map function, which checks if each record contains (or not) the indexed attribute value. The Map function outputs a set of key-value pairs corresponding to primary keys and boolean values. The Partitioner function distributes the key-value pairs evenly across the n reducers (i.e., partitions). The Grouping and Sorting Comparator function groups and sorts, respectively, the key-value pairs in order to form blocks of size m within each partition. Finally, the Reduce function receives the key-value pairs grouped according to the block size. Moreover, each Reduce tasks corresponds to a different partition. The Reduce function processs each block of key-value pairs emitting the first fact table primary key pk_F as key, and the block bitmap array as value. The bitmap partitions are stored as Sequence Files in the HDFS.

Appendix B. Bitmap Star-join Processing Algorithm in MapReduce

The MapReduce implementation of the star-join processing is detailed in Algorithm 4. The algorithm consists of two MapReduce jobs: the first processes the bitmap indices and access the fact table; the second access dimension tables and performs the join operation. In the first job, the Map function reads the bitmap indices, returning key-value pairs corresponding to primary keys and bitmap blocks. The Reduce function processes blocks with the same key. It executes the logical bitwise operations according to the predicates of the query Q (line 1). Then, it generates a list of primary keys (lines 2-8). Lastly, it returns the result of the random access of the fact table (lines 9-12). In the second job, the Map function process the result of the first job (line 2), together with the dimension tables (lines 4-5). The mapping of the dimension records is defined according to a parameter p, which computation is detailed by Afrati et al. [9]. The Partitioner function combines the primary keys and the mapping parameter values to define to which Reduce task a record must be sent. The Reduce function performs the join operations with the help of hash maps structures (lines 2-8).

Appendix C. Bitmap Star-join Processing Algorithm in Spark

The Spark implementation of the star-join processing is detailed in Algorithm 5. The algorithm starts by loading the bitmap arrays in RDDs (lines 1-3). Then, logical bitwise operations are executed according to the predicates of Q(lines 4-7). The RDD generated in the last step is used to build a partitioned list of primary keys from the fact table (line 8). This list is passed to a BulkGet function that randomly accesses tuples from the fact table (line 9). Next, the dimension tables are read and filtered, generating a set of RDDs (lines 9-12). The next step consists in joining the resulting RDDs, which depends on the join strategy chosen. The *SP-Broadcast-Bitmap* performs a hash join by broadcasting the dimension RDDs to all nodes (lines 14-17), while the *SP-Bitmap* executes a sequence of joins between the dimension and fact RDDs (lines 19-22).

Algorithm 3 Creation of the dBJI in MapReduce

input: F, D, a, $value$, t, n and m

F: fact table

D: dimension table

a: indexed attribute

$value$: indexed value of a

t: number of tuples of the fact table

n: number of reducers (equal to number of bitmap)

m: number of tuples indexed in each bitmap block partitions

output: a join bitmap index representing $a = value$

Map Setup

H is a hash map to store the filtered dimension table

1: $Result$ = ReadDimensionTable(D, $a = value$)
2: $H.add($ pk_D from $Result$)

Map(*k*, *v*)

k is null
v is a record from F

1: **if** $H.has($ fk_D from *v*) **then**
2:     Emit (pk_F, 1)
3: **else**
4:     Emit (pk_F, 0)
5: **end if**

Partitioner(*k*)

k is the value of pk_F

1: Return $\frac{k * n}{t}$

Grouping/Sorting Comparator(k_1, k_2)

k_1 and k_2 are two values of pk_F being compared
to compose blocks within partitions

1: Return $\frac{k_1}{m} < \frac{k_2}{m}$

Reduce(*k*, *v*)

k is the value of pk_F stored at the beginning of each block
v is 0 or 1

1: $Bitmap \leftarrow$ []
2: $i = 0$
3: **for each** *value* in *v* **do**
4:     $Bitmap[i]$ = *value*
5:     $i+ = 1$
6: **end for**
7: Emit (*k*, $Bitmap$)

Algorithm 4 Bitmap Star-Join Processing in MapReduce

input: Q, F, D and BJI

Q: star join query

F: fact table

D: set of dimension tables

p: set of mapping parameter values

BJI: set of bitmap join indices

output: result of Q

First MapReduce Job

input: BJI

output: tuples from fact table

Map(*k*, *v*)

k is the value of pk_F of the first tuple indexed by the bitmap array

v is a bitmap array

Emit (*k*, *v*)

Reduce(*k*, *v*)

k is the value of pk_F of the first tuple indexed by the bitmap arrays

v is a set of bitmap arrays with the same pk_F

$Bitmap$ = BitwiseLogicalOperations(*v*)
$KeyList \leftarrow \emptyset$
for $i \in \{0, ..., Bitmap.length - 1\}$ **do**
    if $Bitmap[i] == 1$ **then**
        $pk = k + i$
        $KeyList \leftarrow pk$
    end if
end for
$Result$ = RandomAcess($KeyList$, F)
for each $tuple$ in $Result$ **do**
    Emit ($tuple$, null)
end for

Second MapReduce Job

input: Q, D and Result from Job 1

output: tuples from fact table

Map(*k*, *v*)

k is null

v is a record from D or result of Job 1

if *v* is from Job 1 **then**
    Emit ([f, $\{fk\}$], $\{m\}$)
else if *v* is from D **then**
    for $i = 0$ **to** $i < p_d$ **do**
        Emit ([d, pk_d, i], $\{a_d\}$)
    end for
end if

Partitioner(*k*, *v*, n)

k is an array of three elements

n is the number of reducers

Return $r = f$(*k*), where $1 \leq r \leq n$

Reduce(*k*, *v*)

k is an array of with the table identifier, primary/foreign keys and attributes

v is a set of records with the same key

H_i are Hash Maps to store data from dimensions

$id = k[0]$
if id is from $\{d\}$ **then**
    H_{id}.add($k[1]$, *v*)
else if {H} **has all** fk in *k* **then**
    for each value in *v* **do**
        Emit ([{H_d.get(fk_d from k)}], *value*)
    end for
end if

Algorithm 5 Bitmap Star-Join Processing in Spark

input: Q, F, D and BJI

Q: star join query

F: fact table

D: set of dimension tables

BJI: set of bitmap join indices

output: result of Q

for each $Bitmap$ **in** BJI **do**
  RDD$_{Bit_i}$ = $Bitmap$.mapToPair(pk_F, $bitmap$)
end for
/* Logical operations of the bitmap arrays */
RDD$_{Bitmap}$ $\leftarrow \emptyset$
for each RDD$_{Bit_i}$ **in** RDD$_{Bit}$ **do**
  RDD$_{Bitmap}$ = RDD$_{Bitmap}$.join(RDD$_{Bit_i}$).mapToPair(pk_F, v_1 op v_2)
end for
/* Creating an RDD with the list of selected row-ids */
RDD$_{keys}$ = paralelize(RDD$_{Bitmap}$.getRowIds())
RDD = BulkGet(F, RDD$_{keys}$)
for each d **in** D **do**
  RDD$_d$ = d
  RDD$_d$.filter(Q).mapToPair(pk$_d$, a_d)
end for
if $joinStrategy$ is *SP-Broadcast-Bitmap* **then**
  for each d **in** D **do**
    H$_d$ = broadcast(RDD$_d$.collect( ))
  end for
  RDD.mapToPair([{H$_d$.get(a_d)}], m)
else if $joinStrategy$ is *SP-Bitmap* **then**
  RDD = RDD$_F$
  for each d **in** D **do**
    RDD = RDD.join(RDD$_d$).mapToPair($fk_{d'}$, [a_d, m])
  end for
end if
